# DNA methylation analysis of murine hematopoietic side population cells during aging

**DOI:** 10.4161/epi.26017

**Published:** 2013-08-15

**Authors:** Oluwatosin Taiwo, Gareth A Wilson, Warren Emmett, Tiffany Morris, Dominique Bonnet, Eugene Schuster, Tomas Adejumo, Stephan Beck, Daniel J Pearce

**Affiliations:** 1 Medical Genomics; UCL Cancer Institute; University College London; London, UK; 2 UCL Institute of Healthy Ageing; University College London; London, UK; 3 UCL Genetics Institute; University College London; London, UK; 4 Cancer Research UK; London Research Institute; Lincoln’s Inn Fields; London, UK; 5 Scientific Support Services; Flow Facility; University College London; London, UK

**Keywords:** hematopoietic stem cells, aging, epigenetics, methylomics, methylome, Nano-MeDIP-seq, DNA methylation, Sdpr polycomb repressive complex -2 (PCRC2), Nav2, Kiss1r, Hsf4

## Abstract

Stem cells have been found in most tissues/organs. These somatic stem cells produce replacements for lost and damaged cells, and it is not completely understood how this regenerative capacity becomes diminished during aging. To study the possible involvement of epigenetic changes in somatic stem cell aging, we used murine hematopoiesis as a model system. Hematopoietic stem cells (HSCs) were enriched for via Hoechst exclusion activity (SP-HSC) from young, medium-aged and old mice and subjected to comprehensive, global methylome (MeDIP-seq) analysis. With age, we observed a global loss of DNA methylation of approximately 5%, but an increase in methylation at some CpG islands. Just over 100 significant (FDR &lt; 0.2) aging-specific differentially methylated regions (aDMRs) were identified, which are surprisingly few considering the profound age-based changes that occur in HSC biology. Interestingly, the polycomb repressive complex -2 (PCRC2) target genes *Kiss1r*, *Nav2* and *Hsf4* were hypermethylated with age. The promoter for the *Sdpr* gene was determined to be progressively hypomethylated with age. This occurred concurrently with an increase in gene expression with age. To explore this relationship further, we cultured isolated SP-HSC in the presence of 5-aza-deoxycytdine and demonstrated a negative correlation between *Sdpr* promoter methylation and gene expression. We report that DNA methylation patterns are well preserved during hematopoietic stem cell aging, confirm that PCRC2 targets are increasingly methylated with age, and suggest that SDPR expression changes with age in HSCs may be regulated via age-based alterations in DNA methylation.

## Introduction

Stem cells have been found in most tissues/organs. These somatic stem cells produce replacements for lost and damaged cells, and it is not completely understood how this regenerative capacity becomes diminished during aging. In addition, diseases of the immune system especially myeloid cancers, inflammatory diseases and immune deficiencies, are major burdens of human aging. As a result, there is remarkable interest to delineate age-based alterations in primitive cells of the hematopoietic system. To study the possible involvement of epigenetic changes in aging of the human hematopoietic system, we used murine hematopoiesis as a model system.

Hematopoietic stem cells (HSCs) are responsible for the maintenance of immune and hematopoietic cells throughout life.^1^ Several studies have reported a decline in HSC functionality and a differentiation bias toward myelopoiesis with age.^2^^-^^6^ This occurs concurrently with in an increase in the absolute number and relative frequency of HSCs.^4^^,^^5^

Primitive HSCs possess high ABC/G2 pump activity and can be identified via Hoechst dye exclusion assays.^7^ HSCs that are identified by their Hoechst exclusion activity are known as side population (SP) cells. We have previously reported that the SP subset of murine HSCs accumulates with age.^4^ In particular, cells with the highest Hoechst exclusion activity (termed “lower SP cells” [LSP]) increase with age when compared with other subsets of murine stem and progenitor cells.^4^^,^^5^ As such, analysis of lower SP cells from aged mice and comparison to lower SP from their non-aged counterparts provides an opportunity to examine the nature of aging murine HSCs that have been identified by a physiological property (ABCG2 activity) that is conserved across multiple species, including humans.^8^

Functional and gene expression changes in aging HSCs have been previously reported.^5^^,^^9^ However, the exact mechanism governing these changes remains to be elucidated. Epigenetic mechanisms have been implicated in regulation of fundamental stem cell functions, some of which are altered during aging of HSCs. Indeed, dysregulated expression of epigenetic modifiers has been reported with age.^9^ DNA methylation (DNAm) involves the addition of a methyl group to the carbon-5 position of cytosine bases, this occurs predominantly in a CG context which is enriched at CpG islands (CGIs), but also at other features in the genome.^10^DNAm in particular may be important in HSC regulation as mice lacking enzymes that catalyze DNAm at CG dinucleotides, DNA methyltransferases Dnmt1, and Dnmt3a, have defects in HSC self-renewal and differentiation.^11^^-^^13^

We have previously described an optimized version of methylated DNA immunoprecipitation-based sequencing (MeDIP-seq)^14^^-^^20^ with low starting concentrations, termed Nano-MeDIP-seq.^21^ Nano-MeDIP-seq is a method that allows the unbiased analysis of genome wide DNA methylation from as little as 50ng DNA.

DNA methylation patterns have been previously reported for young HSCs, and more recently DNA methylation patterns analyzed via bisulphite sequencing have been reported for aging HSCs.^22^^-^^24^ Here, we utilized Nano-MeDIP-seq for the analysis of the SP-HSC methylome and report global DNA methylation analysis of a cell population that is highly enriched for HSCs throughout murine aging.

## Results

### Isolation of Lower Side Population cells

We isolated lower side population cells (LSPs) via flow cytometry from adult female C57b/6 mice at different time points: Young (8 – 12 weeks, referred to as Young), middle-aged (~12 mo, referred to as Mid), and old (22 - 24 mo, referred to as Old). Figure 1A shows the sample collection logic for this study. We enriched for HSCs by selecting for LSPs, which are bone marrow cells defined by the lowest Hoechst staining as determined by their position on the nucleated bone marrow cells (NBMCs) Hoechst profile plot (Fig. 1B). We confirmed, by antibody staining for canonical stem cell markers that are reportedly conserved during the murine lifespan (Lin^-^, c-Kit^+^, Sca-1^+^ (KLS); and CD150^+^) that LSP cells isolated in this study, (referred to from this point on as SP-HSCs) were enriched for HSCs (Fig. 1C).^3^ Further confirmation of the immature phenotype of sorted cells was achieved via re-analysis of previously isolated cells for c-Kit^+^, Sca-1^+^, CD150^+^, and CD48^-^ (Fig. 1D, murine cells analyzed in a carrier human cell line [KG1a]).

**Figure:**
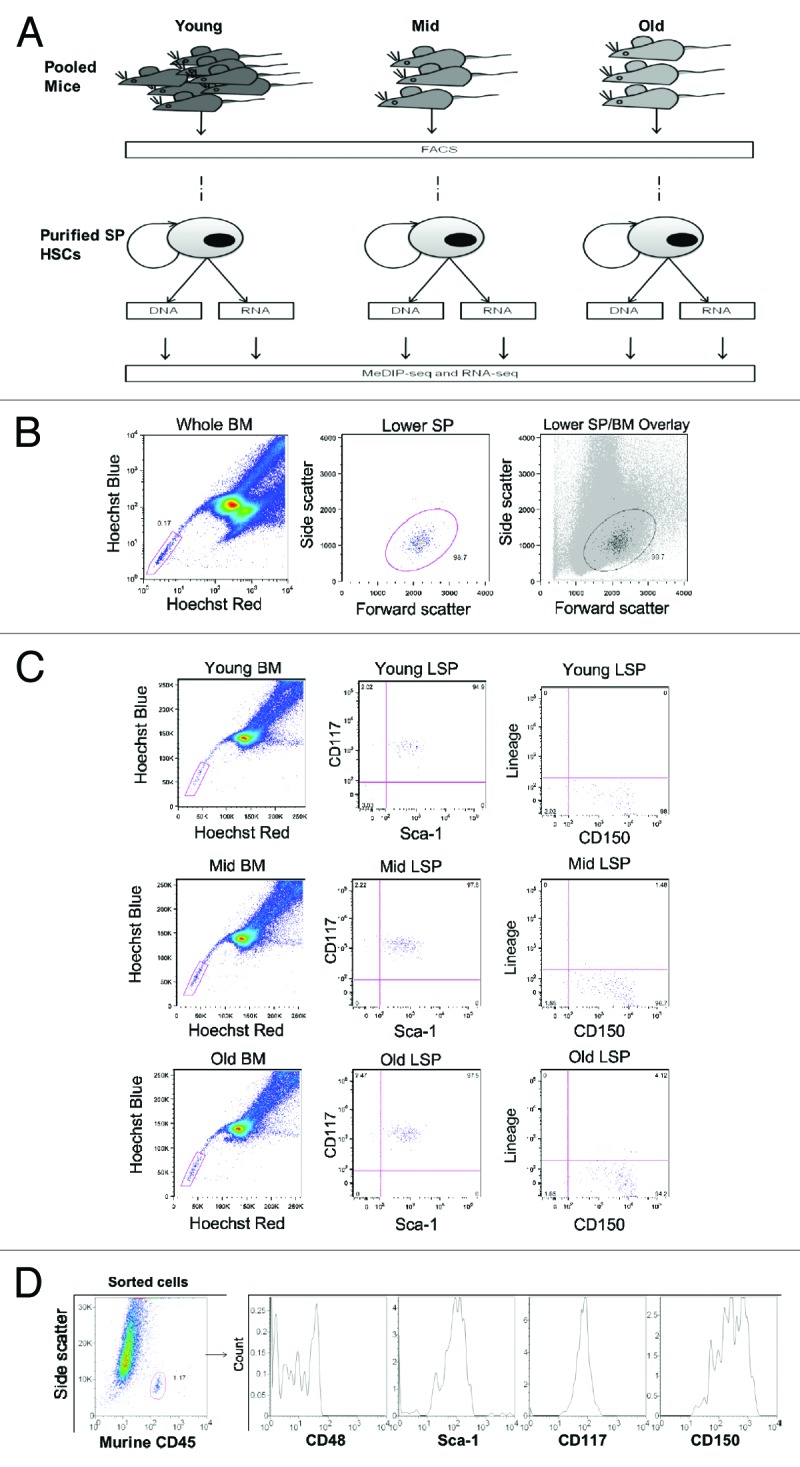
**Figure 1.** Isolation of LSP cells. (**A**) Schematic of study design. SP-HSC were isolated from C57bl/6 mice at three time points from pools of 30 – 45 mice and 3 - 10 mice for each replicate of Young and Mid/Old samples respectively. DNA and RNA was concurrently isolated from FACs purified SP-HSC. 150 - 300 ng of DNA was used for methylome analysis by MeDIP-seq and 50 – 100 ng RNA was used for transcriptome analysis by RNA-seq. Three biological replicates were performed for each step as indicated by (x3). (**B**) Side population (SP) analysis of Hoechst stained bone marrow cells by FACs. Cells were first gated to select for live cells and Hoechst staining (left), to select for lower SP (LSP) cells that show uniformity in their scatter profile (middle and right). (**C**) Confirmation that LSP cells from young, mid, and old mice are enriched for LT-HSCs, by their positivity for CD117 (c-Kit), Sca-1, and CD150, as well as negativity for lineage antigens. (**D**) Confirmation of the immature phenotype of isolated LSP cells via staining of sorted cells for CD48, Sca-1, CD117, and CD150.

### Generation of the SP-HSC methylomes and data quality assessment

We obtained DNA for three separate pools at the aforementioned three time points and generated the corresponding SP-HSC methylomes in triplicates for each age group, using Nano-MeDIP-seq (Fig. 1A). This resulted in an average of 5.25 Gb raw paired-end reads per samples (SD ± 1.15 Gb) of which approximately 90% were successfully paired and aligned to the mouse genome (NCBIM37). After filtering (materials and methods), we obtained an average of 1.92 Gb (SD ± 0.3 Gb) high quality (q ≥ 10), uniquely mapping paired-end reads. Approximately 60% of all CpGs in the mouse genome were covered at least 1 fold (Fig. S1), and all MeDIP samples showed a clear CpG enrichment when compared with input control samples (Average MeDIP enrichment score = 2.73; SD ± 0.12; n = 9, Average input enrichment score of 1.11; SD ± 0.03; n = 2). We found a good correlation for all SP-HSC MeDIP samples (average Pearson’s correlation of 0.84, SD ± 0.09). One of the biological replicates for the “Old” time point had a lower correlation of approximately 0.7 and was excluded from global analyses to maintain stringency.

### Analysis of the aging SP-HSC methylomes

To investigate DNAm changes in SP-HSC with age, we performed comparative analyses (materials and methods) on normalized methylome data from the Young, Mid, and Old SP-HSC. We detected a global loss of DNAm with age (Fig. 2A) which we quantified to an approximately 5% decrease (p < 0.001, Kolmogorov-Smirnov test) in the average methylation level aggregated over all covered CpG sites in the Old compared with Young samples (Fig. 2B). We did, however, observe an age-related gain of DNAm at some CGIs in Old samples (Fig. 2C).

**Figure:**
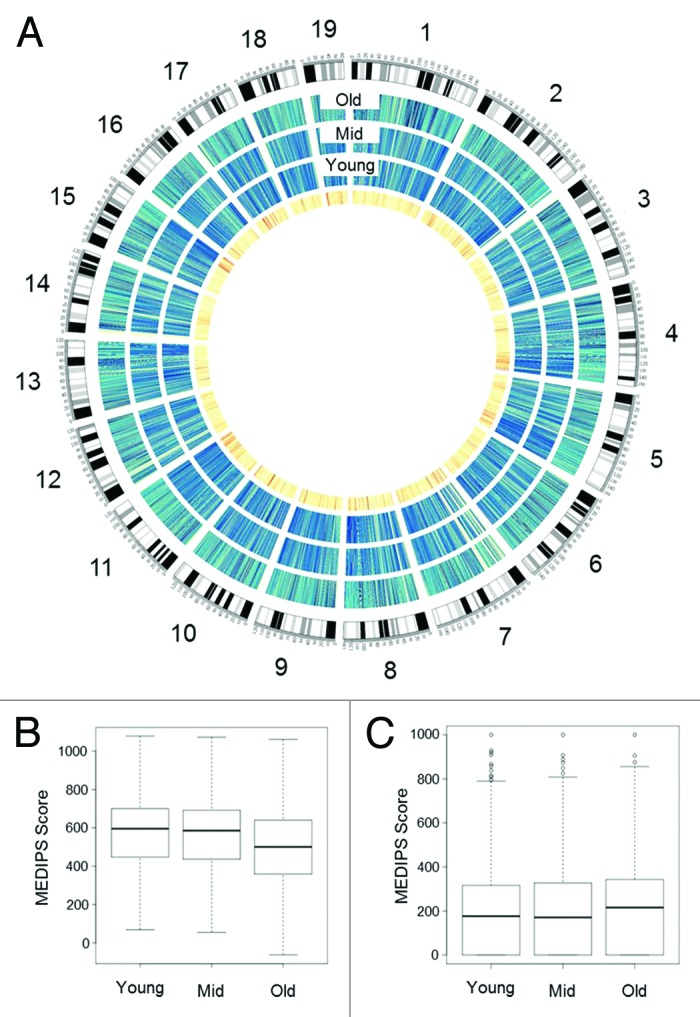
**Figure 2.** HSC Methylome. (**A**) Circos plot of SP-HSC methylomes. Outermost circle shows an ideogram of the mouse genome subdivided by chromosomes. The three blue circles show the Old, Mid, and Young SP-HSC methylomes, respectively. The color code indicates the level of global methylation (dark blue, high, and light blue low). The innermost circle depicts CpG density (dark orange, high, and light orange low). (**B**) Box plots of global and (**C**) CGI, DNA methylation levels for Young, Mid, and Old SP-HSC. DNA methylation levels are expressed as MEDIPS scores.

The MeDUSA pipeline^20^ was used to determine age-specific differentially methylated regions (aDMRs) between the different time points. 53.8% of the mappable mouse genome had a minimum of 10 reads, which allowed us to confidently detect the presence of aDMRs between our Young, Mid, and Old methylomes. A total of 111 significant (FDR < 0.2) aDMRs were found between Old and Young samples, of which 71 were hypermethylated and 40 hypomethylated in the Old samples. These aDMRs appeared to be randomly distributed across all mouse chromosomes (Fig. 3A), with the exception of chromosome 14 and 16 (where no DMRs were detected) and chromosome 5, where we found a hotspot significantly (p-value < 0.001) enriched for significantly (FDR < 0.01) hypermethylated aDMRs. This age-related methylation hotspot corresponds to the location of at least 4 known members of the *Speer* gene family, which includes 14 genes that encode putative glutamate-rich proteins of unknown function.

**Figure:**
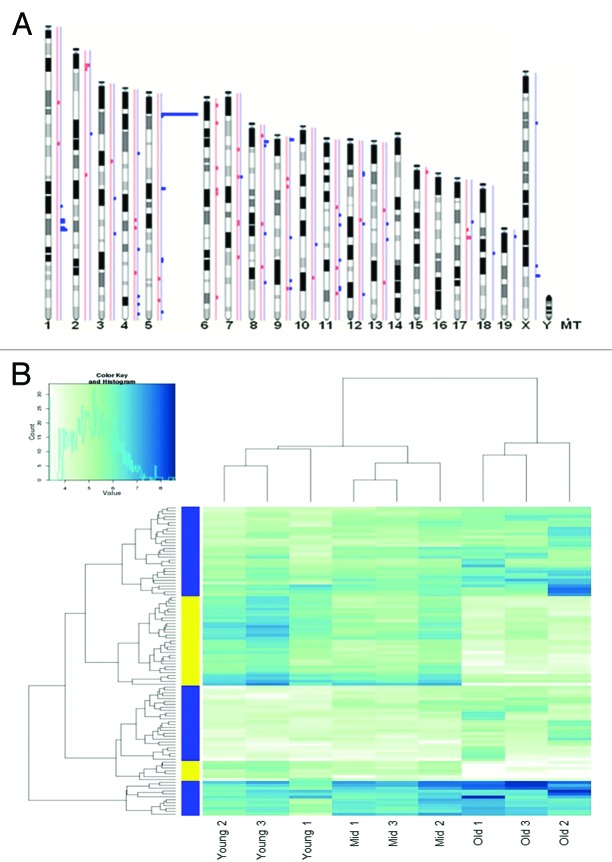
**Figure 3.** Differentially methylated genes. (**A**) Location of significant (FDR < 0.2) age-specific differentially methylated regions (aDMRs). aDMRs were mapped to corresponding genomic coordinates on mouse chromosomes and are represented on an Ensemble-generated ideogram (Ensemble, mm9 genome build NCBIM37). Hyper aDMRs are shown in blue and hypo aDMRs in red. (**B**) Methylation heatmap of all aDMRs. Yellow indicates hypomethylation and blue indicates hypermethylation.

Using a previously determined cut-off of 100 Kb,^25^ we were able to associate 97% of the identified aDMRs with genes, henceforth referred to as differentially methylated genes (DMGs) (Table S1), and observed a progressive change in methylation with age for many aDMGs. Figure 3B shows a DNAm heatmap for all DMRs, displaying progressive and directional DNAm change at several regions in the Young, Mid, and Old samples.

Using annotation from Ensembl (Ensembl 62), aDMRs were categorized according to their genomic location. Hypermethylated aDMRs were significantly enriched in CGIs (Fishers Exact p-value = 2.33E-09), CGI shores (p-value = 4.30E-09), and exons (p-value = 6.18E-11), and hypo aDMRs were significantly enriched in exons (p-value = 2.14E-06), CGI shores (p-value = 8.39E-05), introns (p-value = 3.6E-03), and intergenic regions (p-value = 0.028). Further analysis using the Ensembl regulatory build (Ensembl 67) revealed that CTCF binding sites were most enriched in the hyper aDMRs and c-MYB sites were most enriched in hypo aDMRs.

### Polycomb repressive complex target genes are differentially methylated with age

It has recently been reported that targets of the polycomb repressive complex -2 (PCRC2) members EED and SUZ12 are differentially methylated with age.^22^Specifically, these PCRC2 targets seem to gain methylation with age. We compared our aDMGs with the data recently published by Beerman et al. and found three PCRC2 target genes that are hypermethylated with age in both studies: *Kiss1r*, *Nav2*, and *Hsf4* (p < 0.0025, Fisher’s exact test).

Examination of aDMRs with histone data from the ENCODE project revealed that *Kiss1r* is hypermethylated with age in the 3′ CGI, an area that has been associated with enrichment of the histone marks H3K4me1, H3K4me3, H3K9ac, and H3K27me3. *Hsf4 *hypermethylation is located in the shore region of its promoter-associated CGI. Interestingly, the region of the Nav2 gene that is hypermethylated with age is intronic, contains multiple regulatory regions (including H3K4me1, H3K4me3, and H3K27ac) and may be a potential enhancer region. In addition to Nav2 hypermethylation, we found an exonic region associated with H3K4me1 that was hypomethylated with age.

Comparison between our data and published data on Suz12 targets in mice ES cells^26^ revealed 2 additional potential targets of the PCRC2, *Amotl2*, and *Cyfip2*, which, in our study, were hyper and hypomethylated respectively. In addition, Trim37 is a PCRC1 target and was hypermethylated with age in our study. One other gene classified as hypo-aDMG in our study (NPAS3) had a functional equivalent that was reported as a PCRC2 target that was hypermethylated with age by Beerman et al. (NPAS2).

### Induced SDPR promoter demethylation is associated with increased gene expression

SDPR has been previously implicated in HSC aging via reports of age-based increases in gene expression.^5^^,^^9^ Here we report that the *sdpr* gene has progressive loss of DNAm with age at the promoter region. (Fig. 4A) This was confirmed by bisulphite pyro-sequencing of independent Young, Mid, and Old samples (Fig. 4B and D). As previously reported, this age-based loss of promoter methylation occurs concurrently with a progressive gain of gene expression in SP cells (Fig. 4C). To further explore this relationship between age-based promoter hypomethylation and increased gene expression, we demethylated highly purified primary SP-HSC by 5-Aza'deoxyCytidine (5-Aza'dC) treatment during a 4 d ex vivo culture process. The culture conditions have been previously described to promote slight expansion (cell division) of HSCs with minimal differentiation.^27^ This treatment protocol resulted in an upregulation of *Sdpr* gene expression (Fig. 4E, p-value = 0.028 for 2 µM treatment). Hypomethylation of the *Sdpr* promoter following 5-Aza'dC treatment was confirmed by bisulfite pyro-sequencing of 2 biological replicates (Fig. 4F, p-value < 0.005). These results are consistent with an inverse relationship between *Sdpr* promoter methylation and gene expression.

**Figure:**
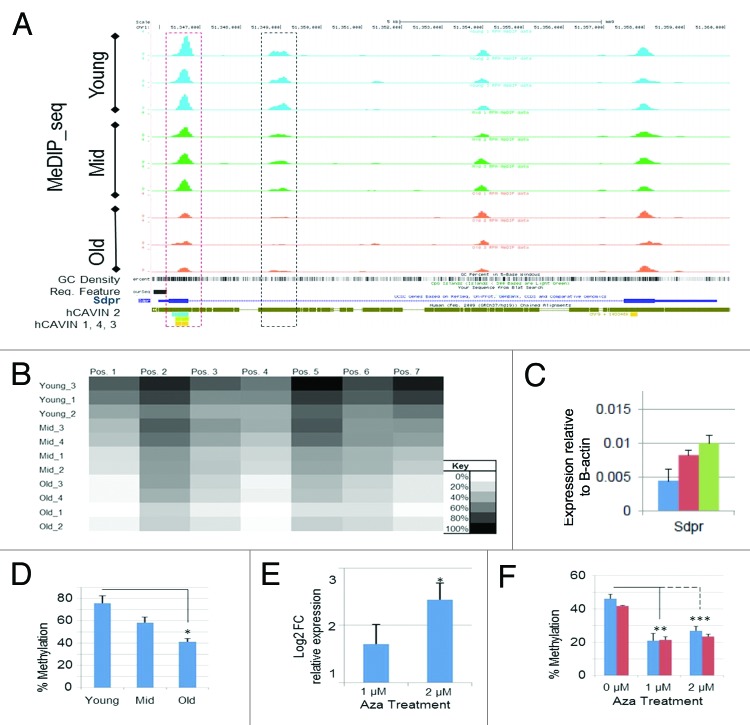
**Figure 4.***Sdpr* analysis. (**A**) Screenshot of MeDIP-seq profiles for *Sdpr* gene in UCSC genome browser (UCSC mm9). Tracks are color-coded blue, green, and orange for Young, Mid, and Old samples, respectively. Three biological replicates are shown for each age group. Peak height represents DNA methylation. The two aDMRs identified in the *Sdpr* gene are boxed in red and black. The red box depicts the aDMR selected for validation (**B–E**). This region aligns with other members of the human CAVIN gene family. ‘Reg. Feature’ denotes the location of the Ensembl promoter associated regulatory feature ENSMUSR00000405109. The promoter associated H3K9ac, H3K36me3, H3K4me3, and RNAP II are localized to this region. (**B**) Validation of *Sdpr* promoter hypomethylation (aDMR boxed in red [**A**]) using bisulfite pyro-sequencing. Data was obtained from SP-HSC pools for three biological replicates for Young samples and four biological replicates each for Mid and Old samples. A methylation heatmap for the individual CpGs assayed, and for each replicate is shown. This data are also displayed as a chart (**D**). (**C**) RT-PCR data to confirm published reports of increased S*dpr* gene expression with age (n = 3 for each, mean ± SD). (**E**) Functional analysis of same *Sdpr *aDMR by *ex-vivo* culturing of SP-HSC in the presence of Aza-deoxycytidine and expression analysis using qRT-PCR. Data was obtained from independent experiments for a total of four biological replicates. Expression values shown are relative to untreated control. (**E**) Validation of same *Sdpr *aDMR for promoter hypomethylation following *ex-vivo* culturing in the presence of Aza-deoxycytidine Data are shown for two biological replicates. 2–3 mice were pooled per replicate and average value for two treatment replicates is shown for each biological replicate (replicate 1 and 2 – blue and red bars respectively). FC is fold change. Error bars depict standard error of the mean (S.E.M) in all cases (*p < 0.05; **p < 0.005; ***p < 0.0005).

## Discussion

We conducted methylome analysis of primitive, primary mouse SP-HSCs from (8–12 weeks), Mid (~12 mo), and Old (22–24 mo) C57bl/6 mice. This population is highly enriched for HSCs^7^^,^^28^ and contains the subset of primitive cells that seems to accumulate with age.^4^ The LSP subset overlaps with other subsets previously demonstrated as enriched for HSCs throughout the murine lifespan.^3^^-^^5^^,^^12^

Consistent with recently published data,^22^ we report that the overall DNA methylation pattern is highly conserved during HSC aging. This is remarkable, especially when one considers the profound changes in HSC biology that occur during murine aging, such as lineage skewing and accumulation of primitive HSC subsets. Despite this overall maintenance of DNA methylation patterns, we report that over 100 genes are differentially methylated with age, the majority of which were defined as hypermethylation.

We also note that when analyzed globally, there is a significant (5%) loss of global DNA methylation during murine HSC aging. This is consistent with studies into human aging of other cell types, but non-HSCs were analyzed.^29^^-^^32^ Our global loss of DNA methylation is however, inconsistent with the HSC global hypermethylation recently reported by Beerman et al.^22^ The most likely reason for this is the use of different technologies. We used MeDIP-seq, which provides approximately 60% genome coverage, including CGIs (where we identified global gain of methylation) and many non-CGI features (where we identified global loss of methylation). Beerman *et* al utilized reduced-representation bisulphite sequencing (RRBS), which only provides 5–10% genome coverage, predominantly of CGIs (where they also identified global gain of methylation) and thus were blind to the much larger loss of methylation at non-CGI features. Where we have overlapping coverage, our data confirm their finding that genes that are targets of the PCRC2 members EED and SUZ12 are differentially methylated with age. In addition, we report age-related differential methylation of 2 other targets of the PCRC2 that were not reported by Beerman et al.^22^

It may be that this age-based methylation of PCRC2 targets occurs as a result of reduced PCRC2 activity with age. Indeed, the PCRC2 complex has been implicated in the control of fundamental HSC cellular functions that may be related to an age-based decline. For instance, HSCs undergo *INK4a/ARF*-mediated senescence when the PCRC2 member *Ezh2* is downregulated.^33^

We chose to examine a possible relationship between *sdpr* gene promoter methylation and gene expression as we observed promoter hypomethylation with age, and an age-related upregulation of *Sdpr* in HSCs has previously been reported.^5^^,^^9^ Three days of 5-Aza'dC treatment on highly purified LSPs resulted in an increased gene expression that occurred concurrently with a reduction in promoter methylation. These results, as well as the findings that *Sdpr* was upregulated in Dnmt1-Knockout (KO) mice HSCs^13^ and in Dnmt3-KO mice HSCs,^12^ is consistent with an inverse relationship between DNAm and *Sdpr* gene expression and suggests that SDPR gene expression may be directly or indirectly controlled by promoter methylation. One caveat is that since 5-Aza'dC is a global demethylation agent, the effect on SDPR expression may be due to demethylation of other genes. However, this proof of principle experiment provides a tool for further analysis of the relationship between DNAm and gene expression in rare subsets of cells enriched for HSCs.

While age-related upregulation of *Sdpr* in HSCs has previously been observed, we have demonstrated that it is linked to loss of promoter methylation. *Sdpr*, which is also known as *Cavin-2*, was originally identified as a gene whose expression was increased in serum-starved cells and may be associated with cellular senescence.^34^ One explanation for the age-based decline in the hematopoietic system that is coupled with an apparent increase in primitive HSCs is an accumulation of damaged or dysfunctional cells that do not contribute to hematopoiesis. It may be that increased *Sdpr* expression in aged HSCs indicates an inactive, senescence-like state.

In addition, an independent role in caveolae formation and cell signaling has been reported.^35^Caveolae are thought to be invaginated lipid rafts that provide a common platform for various signaling molecules, leading to enhanced cell signaling and potent signal transduction. Overexpression of *Sdpr* in cultured cells leads to caveolae elongation^35^ and perhaps results in an increased surface area for interacting cell signaling molecules. Increased expression in aged HSCs may cause an altered sensitivity to survival or growth signals.

In conclusion, we report that DNA methylation patterns are well preserved during hematopoietic stem cell aging, confirm that PCRC2 targets are differentially methylated with age, and suggest that SDPR gene expression changes with age in HSCs may be regulated via age-based alterations in promoter DNA methylation.

### Materials and Methods

### Mice and hematopoietic stem cell preparation

Wild-type Young (8–12 weeks), Mid (~12 mo), and Old (22–24 mo) C57bl/6 mice were sacrificed using an approved method. Mice were originally from the Jackson immune-research laboratories (Bar Harbour, ME). All of our animals were maintained in a specific pathogen-free animal facility. Whole bone marrow cells were isolated by gentle flushing into 1x HBSS, 10mM Hepes solution (Sigma Aldrich) using a 27G syringe. Erythrocytes were lysed with 3 volumes of Ammonium Chloride (Stem Cell Technologies) and debris removed by filtering cell suspension through a 40 µM nylon cell strainer. SP staining was performed as previously described.^7^ Briefly, total nucleated bone marrow cells (NBMCs) were resuspended in DMEM staining media at 1 × 10^6^ cells per ml and incubated with 5 µg per ml Hoechst 33342 DNA binding dye (Sigma Aldrich) for 90 min at 37°C with mixing at 30 min intervals. Hoechst stained cells were resuspended in DMEM, 2% FCS, and 10 mM HEPES solution at approximately 1 × 10^7^ cells/ml. Cells were maintained at 4°C until analysis/sorting and during antibody labeling. Lower SP cells were sorted directly into 1.5 ml eppendorf tubes containing either 1x PBS, or RLT cell lysis buffer (Qiagen) for single RNA extraction or Stemspan Serum-Free Expansion Media (SFEM, Stem Cell Technologies) for *ex-vivo* culture. Approximately 5 × 10^6^ Hoechst stained NBMCs were analyzed for HSC immunophenotype by SP;KLS;CD150,CD48. Antibody labeling was performed as previously described^4^ using the following antibodies: CD150, phycoerythrin (PE, Biolegend); Sca-1, PE/Cyanine7 (PE/Cy7, Biolegend); CD 117 (c-Kit),allophycocyanine/Cy7 (APC/Cy7, eBioscience); and Biotinylated Lineage antibody cocktail (anti-CD5, anti-CD11b, anti-B220, anti–Gr-1, and anti–Ter-119), subsequently labeled with Percp-conjugated streptavidin (SA-Percp, BD Biosciences). An amount of 2 µg/ml Propidium iodide (PI, Sigma Aldrich) was added to resuspension media to facilitate the exclusion of dead cells during cell sorting on the Moflo cell sorter (Beckman Coulter) and NBMCs analysis on the LSRII (BD Biosciences). PI was substituted for 1:1000 Dapi (Sigma Aldrich) for post sort analysis on the Cyan ADP (Beckman Coulter).

### Ex vivo culture and aza-cytidine treatment of primary SP-HSCs

Lower SP cells were purity sorted into SFEM and cultured as previously described^27^ but with minor adjustments to cytokine concentrations. Cells were incubated at 37°C and 5% CO_2_, in a round bottom 96 well plate (corning) at 1000–2000 cells per 200 µl SFEM plus 2 µg Heparin and cytokines (3 ng recombinant mouse SCF, 6 ng recombinant mouse TPO, 6 ng recombinant mouse IGF-II, 3 ng recombinant human FGF-I, and 30 ng recombinant mouse ANGPTL3). For 5-Aza'dC induced hypomethylation, ex vivo cultured cells were treated with two doses of 0–2 µM 5-Aza'dC. The first dose was applied approximately 18 h after culture was initiated. Cells were maintained for a further 30 h after which 50% of media was replaced with fresh 100 µl SFEM plus 1 µg Heparin and 33.3% of the original cytokine concentrations listed above. 50% of initial 5-Aza’dC dose was added to cells 18 h after media replacement and ex vivo culture was continued for another 30 h. LSP cells were cultured ex vivo for a total of 4 d.

### DNA isolation and methylation analysis

An amount of 150–300 ng of DNA was isolated from 7–8 × 10^4^ LSP cells in RLT lysis buffer. Thirty to forty Young mice and 3-10 Mid, or Old mice were pooled per sample. DNA and RNA were simultaneously extracted from pooled LSPs using the AllPrep DNA/RNA Kit from Qiagen according to manufacturer’s instructions. Methylation analysis of extracted DNA was performed by Nano-MeDIP-seq as previously described.^21^MeDIP libraries of 190–200 bp was prepared for three biological replicates each for 2, 12, and 24 mo old mice and subjected to 36 bp paired end (PE) sequencing on either the Illumina GA IIx (“Old 1,” “Old 2”, and “Young 1”) or the Illumina Hi-seq platform for all other samples. Pyro-sequencing was used for validation. Briefly, 1 × 10^4^ cells per sample were bisulfite converted using EpiTect Plus LyseAll Bisulfite Kit and bisulfite conversion efficiency was found to be at least 99% for all samples as determined by qPCR. Control reactions on unmethylated and in vitro methylated samples were also conducted to confirm a linear detection of methylation levels by pyro-sequencing. Pyro-sequencing and PCR amplification of candidate regions were conducted using the PyroMark assay design software and PyroMark PCR kit respectively. Pyro-sequencing was performed using the PyroMark Gold Q96 reagents and the PyroMark Q96 MD Pyro-sequencer. All materials used for Pyro-sequencing were from Qiagen, except for primers, which were obtained from Sigma. All methods were performed according to manufacturer’s instructions.

### RNA purification and transcript analysis

Differential expression of candidate genes was analyzed by quantitative reverse transcriptase- PCR (qRT-PCR). Here, 0.5–1 × 10^−4^ cells per sample were converted to cDNA using the SuperScript® III CellsDirectcDNA Synthesis Kit (Invitrogen). qRT-PCR of individual transcripts was then performed using Taq-Man gene expression assays (Applied Biosystems). All steps were completed according to manufacturer’s instructions. Gene expression was normalized to β-Actin housekeeping gene and fold change (FC) was calculated by the ΔΔCt method.

### MeDIP-seq data analysis

The generated MeDIP-seq data were analyzed using our computational pipeline MeDUSA^20^ v1.0.0 and the MEDIPS^20^^,^^36^ v1.0.0 R Bioconductor package. MeDUSA constitutes several discrete stages of analysis. Briefly, BWA v0.5.8^37^ was used to align the paired end sequence data to the reference mouse genome build (NCBIM37) using default settings. Filtering was performed to remove reads failing to map as a proper pair and those pairs in which neither read scored an alignment score of ≥ 10. In cases of non-unique reads, all but one pair was removed. Quality control was performed using the tool FastQC v0.9.4 (http://www.bioinformatics.bbsrc.ac.uk/projects/fastqc/) and MEDIPS. Additionally between replicate genome-wide correlations were calculated using QCSeqs from the Useq package.^38^ Correlations were calculated using a window size of 500 bp, increasing in 250 bp increments. A minimum number of 5 reads in a window was required prior to inclusion in the correlation. DMRs were called using the Bioconductor package DESeq.^39^DESeq was run with the estimateDispersions sharing mode set as ‘fit-only’. Only regions containing a minimum of 10 reads summed from the cohorts being compared were included for DMR analysis (termed our bump-list).

In order to identify regions of the genome enriched for DMRs, permutation analysis was performed. Our DMRs were placed in 100 kb windows across the genome and the most enriched location was identified and the relevant DMR count stored. 111 regions were randomly selected from the bump-list and placed in 100 kb windows. The windows were assessed to determine how often a region was found greater than our observed data. This was performed 1000 times in order to calculate an empirical p-value for our region of interest.

Genome-wide methylation scores were generated using the MEDIPS package. The paired reads were not extended and were placed in bins of 50 bp across the genome. The CpG coupling vector was generated with fragment lengths of 700 bp and using the “count” function. Circos v0.55^40^ was used to generate a circular genome plot displaying these methylation scores. Feature annotation was obtained from Ensembl 62 and from the Ensembl Regulatory Build 67. CpG island shores were defined as extending 2000 bp upstream and downstream of an annotated CpG island. DMRs and bumps were placed in their relevant features using custom perl scripts and bedtools^41^ v2.10.2. We used the Fishers Exact test to identify feature types enriched for DMRs.

### Accession Numbers

The generated MeDIP-seq data was deposited into the GEO database under accession number GSE41658 .

## Supplementary Material

Additional material

## Abbreviations:

HSC: hematopoietic stem cell
SP: side population
LSP: lower side population
SSCs: somatic stem cells
NBMCs: nucleated bone marrow cells
DNAm: DNA methylation
CGI: CpG island
MeDIP: methylated DNA immunoprecipitation
MeDIP-seq: methylated DNA immunoprecipitation based sequencing
DMR: differentially methylated region
DMG: differentially methylated gene
aDMR: age differentially methylated region
qRT-PCR: quantitative reverse transcriptase polymerase chain reaction
SFEM: Serum-Free Expansion Media
Aza or 5-Aza’dC: 5-Aza’deoxyCytidine
FDR: false discovery rate
S.D: standard deviation
FC: fold change
PCRC2: polycomb repressive complex -2
