# Supplementary material for: DNA methylation analysis of murine hematopoietic side population cells during aging
Source: Epigenetics. 2013 Aug 15;8(10):1114–22. doi: 10.4161/epi.26017 (PMC3891692; doi:10.4161/epi.26017)
Supplement: Additional material [file epi-8-1114-s01.pdf]

Landes Bioscience www.landesbioscience.com

Supplemental Material to:

Oluwatosin Taiwo, Gareth A Wilson, Warren Emmett, Tiffany Morris, Dominique Bonnet, Eugene Schuster, Tomas Adejumo, Stephan Beck, and Daniel J Pearce

DNA methylation analysis of murine hematohematopoietic side population cells during aging

Epigenetics 2013; 8(10)
http://dx.doi.org/10.4161/epi.26017

www.landesbioscience.com/journals/epigenetics/article/26017

Supplementary data files

Figure S1. CpG coverage plots for Young (Y 1-3), Mid (M1-3) and Old (O 1-3) SP-HSC methylomes: Approximately 60% of all CpGs in the genome were covered at least once and 24-33% were covered at least five times.

Table S1. List of genes associated with aDMRs are shown with FDR scores for: **(A)** hyper aDMRs and **(B)** hypo aDMRs. FDR scores ≥ 20, ≥ 13, ≥ 10 and ≥ 7 correspond to false discovery rates of ≤ 0.01, ≤ 0.05, ≤ 0.1 and ≤ 0.2 respectively.

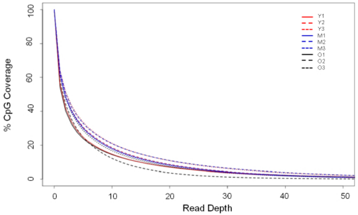

% CpG Coverage
Read Depth
0
20
40
60
80
100
10
30
50
Y1
Y2
Y3
M1
M2
M3
O1
O2
O3

Table S1

A

FDR Score	**Associated Gene (Hypermethylated)**
126.3403094	Gm10719
75.43196226	Speer7-ps1
54.6491772	Gm17019
47.01435918	Speer7-ps1
37.33760368	Speer4e
36.79079843	Obscn
32.89293998	Ppfia4
29.10987819	Gm10354
28.42895091	Kiss1r
28.02147349	Speer4e
27.34598614	Nhlh2
26.24206324	B230323A14Rik
25.86843874	Steap3
24.43467347	SNORA17
21.5311003	Speer4e
21.23280692	Gm17019
21.**05836842**	Iscu
20.91274887	N/A
20.48887312	Fmod
19.31867144	Speer7-ps1
19.11426509	Gm11167
17.61612833	Gm16512
17.55861097	Thsd7b
17.2388252	Gm17019
16.27777594	Ltbp3
15.6720591	Ptrprv
15.2637275	9030624G23Rik
14.92995941	Speer7-ps1
14.49169005	Gm17699
14.08237908	Speer4e
14.00703296	Rundc3a
13.79534053	Kcnab2
12.15210127	N4bp3
12.13526114	8030443G20Rik
12.06944071	Nav2
11.66153895	Nr4a2
11.66153895	SNORA17
11.58800333	Rnf8
11.44098632	4930572O03Rik
11.38917302	Gsx1
11.31542986	2410018L13Rik
11.2480951	Aldoart2
10.6544988	Speer4f
10.38528526	Nav1
10.27772**615**	Rusc2
10.09287214	Gm10354
10.05293032	Hsf4
9.945993896	Amotl2
9.862435173	Upf3b
9.862435173	SNORA17
9.858895227	Atg4a
9.719965993	SNORA17
9.587078734	Commd10
9.526353902	AC163338.1
8.87133761	N/A
8.706436836	Insl3
8.701147058	Atg4a
8.449773491	Thsd7b
7.953359713	Gm13017
7.915029128	Prima1
7.818868024	Gm9758
7.818868024	Gm12575
7.737277286	Dlgap2
7.651509828	Smarca5-ps
7.620455596	Drp2
7.346187707	Lmod1
7.215984188	Sez6l
7.208128242	Tubg2
7.175549618	4930572O03Rik
7.11816745	Gm10354
7.022499062	Gm17699

B

FDR	**Associated Gene (Hypomethylated)**
158.8895	Trim30a
40.91621	Cftr
35.64826	N/A
35.59651	Utp11l
25.83163	Slc39a12 or Mrc1
24.4732	Trim37
24.24417	U6
23.12683	Ptger4
20.46584	Gm15821
20.2128	Dcps
19.11427	mmu-mir-2133-2
17.9525	Gm10663
15.94277	Pde8b
14.26289	Gm13295
13.86083	Pfkfb3
13.18236	Smtnl1
13.11545	Itgb3
11.79247	Tnip3
11.66154	Wdfy1
11.661**54**	Mir715
11.65005	Cyfip2
11.566	Nav2
10.98418	Iqgap1
10.82393	Cpne2
10.67865	Gm17028
10.26196	Gm16206
10.21662	N/A
10.14504	Slco2a1
9.859645	Gm17247
8.932791	Npas3
8.922945	Sdpr
8.591598	Mtap
8.585563	1600014C10Rik
8.297465	Rilpl2
8.214479	Gm12648
7.907489	Slc25a13
7.90583	Loh12cr1
7.371706	Cul1
7.369538	Atxn1
7.230497	Pde8a
